# Book Reviews

**Published:** 1806-04

**Authors:** 


### CRITICAL ANALYSIS

OF THE

### RECENT PUBLICATIONS

ON THE

### DIFFERENT BRANCHES OF PHYSIC, SURGERY, AND MEDICAL PHILOSOPHY.

---

*The History and Treatment of the Diseases of the Teeth, the Gums, and the Alveolar Processes, with the Operations which they respec-tively require; to which are added, Observations on other Diseases o the Mouth, and on the Mode of fixing artificial Teeth. Illustrated with Copper-plates.* By JOSEPH FOX, Member of the Royal College of Surgeons, and Lecturer on the Structure and Dis-eases of the Teeth, at Guy's Hospital. Quarto, pp. 175, London, 1806.

THE first part or volume of this work, which contains the natural history or physiology of the human teeth, and the proper mode o treatment to prevent irregularities during the second dentition, &c. was noticed by us in a former number.* That volume, althoug it contained an account of the diseases which affect children dur-ing the first dentition, yet it advanced no further than the com-pleat formation and regularity of the second set of teeth. Th present work commences with the first appearances of decay an disease, and contains the whole pathology of these organs; so that together, they comprehend the substance of the lectures delivere by the author at Guy's Hospital.

On the appearance of the first volume, our author's opinion, respecting the vascularity and constitutional vitality of the teeth met with some slight opposition. As he did not think it necessar to take any public notice of it at that time, he adverts to it in th Introduction to this volume.

"In my former Treatise," says he, "I endeavoured to prov that the teeth are organized in a similar manner to other bones and that, as possessing life, they are connected with, and form a integral part of the system. Since its publication, I have had th satisfaction to find, that the same opinion is entertained by almos all the enlightened members of the surgical profession."

This point of physiology was necessary to be settled, as withou it, we could not in any satisfactory manner assign a cause for th different diseases of the teeth. These diseases are similar to thos which affect bones in general, and in like manner they have thei origin in inflammation. The teeth differ only from bones in no possessin

---

* See Medical and Physical Journal for July, 1803, p. 77.

possessing sufficient living power to effect the process of exfoliation. These different kinds of diseases affect various parts of the teeth. *Caries* is the death of any part of the crown of the tooth; but the fangs, the membrane contained within the cavity of a tooth, the enamel, the gums, and the *alveoli* or sockets, have all their distinct diseases, as well as others in common with each other, and they are moreover liable to be affected by diseases of the constitution.

In addition to these various diseafed actions, there is an earthy depofit, called tartar, which, in a greater or lefs degree, accumulates about the teeth of moft perfons; this, if fuffered to increafe to any quantity, caufes a feparation of the gums from the necks of the teeth, and a confequent abforption of the alveolar proceffes.

There are alfo many difeafes to which the *antrum maxillare* is liable; and different imperfections of the palate or roof of the mouth, arifing either from natural conformation, or the confequence of difeafe; and after thefe fubjects have been duly confidered by our author, the mode of fupplying the defect attending the lofs of teeth, by means of artificial ones, is defcribed; and an account of the method of performing the feveral operations which the difeafes of the teeth require, concludes the work.

Agreeably to this fketch, the book is divided into three parts, treating of the difeafes of the *teeth*, of the *gums*, and of the *manner of performing the operations*.

In the firft of thefe are difcuffed the following fubjects, viz. Caries, exoftofis of the fangs, necrosis affecting the teeth, fpina ventofa, removal of the enamel, wearing of the teeth by maftication, and fractures of the teeth.

In the fecond part are treated, gumboils, fcurvy in the gums, preternatural growth of the gums, difeafes of the alveolar proceffes, the tartar of the teeth, the analyfis of the tartar by Mr. Pepys; the refult of which is,

| | |
|---|---|
| Phofphate of lime - - - - | 35 |
| Fibrina, or cartilage - - - - | 9 |
| Animal fat, or oil - - - - | 3 |
| Lofs - - - - - - - - - - - | 3 |
| | 50 |

The effects of mercury upon the teeth. This very important part of the fubject is treated by our author at confiderable length, and cannot fail to be read with great intereft. The fame miferable confequences which arife from the improper ufe of mercury, often follow the fmall-pox, of which fome inftances are produced. The next fubject, is the *antrum maxillare* as the feat of difeafe, the imperfections in the palate; and laftly, we have a general ftatement upon the fubject of artificial teeth.

In the third part, the following operations are treated of, viz: filing the teeth, ftopping the teeth when hollow, the mode of applying plying ligatures to the teeth, fcaling the teeth, extraction of th e teeth, and luxation of the jaw.

All thefe important fubjects are treated by Mr. F. in the fame perfpicuous and accurate manner, which characterized the firft volume. And as the great object of the author appears to be that of furnifhing his profeffional brethren with a correct practical work, on a fubject which has been the conftant object of his attention from his youth, in which he has been engaged as a fuccefsful practitioner in the centre of the metropolis, and on which he has lectured with approbation for many years, he has availed himfelf of the aid of the engraver. We believe Mr. F. is the firft author who has employed thefe means of depicting the *difeafes* of the teeth. Every one knows how much plates, when accurately executed, as is the cafe here, contribute to elucidate practical fubjects. In this work, we are convinced that every reader will acknowledge, that arrangement and perfpicuity of language, combine with the aid of engravings, to remove all doubt or obfcurity on this interefting fubject. As we could not give the plates, we have declined the infertion of partial extracts, and refer our readers with great fatisfaction, to the book itfelf.

---

*Reply to Dr. James Carmichael Smyth, containing Remarks on his Letter to Mr. Wilberforce; and a further Account of the Discovery of the Power of Mineral Acids in a State of Gas to destroy Contagion.* By John Johnstone, M. D. Fellow of the Royal College of Physicians, &c. 8vo. pp. 275. London: 1805.

Dr. J. commences his Reply by the following concise statement of dates and facts, which appear to be admitted on all sides.

"It is not be denied," says he, "that the first public mention of the respirable mineral acid gases, as correctors of putrefaction, was made by Dr. James Johnstone, sen. late physician in Worcester, in 1758. The second mention of them was made by M. Guyton de Morveau in 1773. In 1779 Dr. James Johnstone, jun. recommended muriatic acid gas, as the surest method to prevent the spread of contagion. In 1795 Dr. James Carmichael Smyth recommended nitric acid gas, to destroy contagion. In 1802 the same Dr. James Carmichael Smyth petitioned the House of Commons to remunerate him, as the discoverer of the power of mineral acids in their gaseous state to destroy contagion. In 180[illegible] I published my account of the discovery, and proved, I hope satisfactorily, the claim of my father to priority of discovery. In 1805 Dr. Smyth, in answer to my pamphlet, published his letter to Mr. Wilberforce."

Dr. S. in his petition, claims the discovery both of principle and agent; Dr. J. admits, that his father employed a different agent, because he preferred the muriatic to the nitric acid, as many practitioners do at present; but contends that his father discovered covered the principle, and that the variation of the agent is of little importance.

When Dr. S. presented his petition to the House of Commons, the health of Dr. J. senior was declining so rapidly, that his son could not attend the Committee to which the petition was referred, but he wrote to Sir William Pultney, to Mr. Addington, and to Mr. Wilberforce. In consequence of the request of the last named gentleman, our author sent the testimonies of his brother and his own, which were presented to the committee. The decision took place; and when our author procured the report of the Committee in August 1782, he wrote his first pamphlet,* intitled " An Account of the Discovery of the Power of Mineral Acid Vapours to Destroy Contagion." This was published in 1803, which produced Dr. S's letter to Mr. Wilberforce in 1805, to which the present work is a reply.

" The main evidence of the date of the discovery is contained in *An Historical Dissertation concerning the Malignant Epidemcial Fever of* 1756, &c. By James Johnstone, M. D. London: printed 1758.

In this Dissertation Dr. J. says, " In treating of the cure of malignant fevers, *I lay down no sanative precept which I have not, in my sphere of practice, experienced abundantly useful, and generally successful.* The cautions which I enter against known practices or remedies are also drawn from a repeated observation of their mischievous effects. Some theoretical reasoning and inferences, however unsuitable to an historical piece, I have added, with a view of rendering the nature of malignant disorders more generally understood than, perhaps, they are by many who will be under the necessity of treating them; and also, further to recommend those rules, of the utility of which *I was primarily convinced by observations,* to those who demand evidence of a different nature. Many practitioners love to know *that a peculiarity in practice is rational, as well as in fact successful,* before they make it their own. In a word, I have thrown together in a narrow compass, and with all possible plainness, several important experimental truths, which I hope may be of service in preparing many, successfully to combat these terrible disorders in situations and circumstances similar to those I have described."

" Such," says our author, " are the declarations of my father concerning his own meaning, and his own work; a work which he avows contains *peculiarity in practice* founded on his own experience, and in which he lays dowu *no sanative precept* unwarranted by its utility and its success in his own practice."

When he comes to treat of the *Cure,* he says, " At the beginning of the fever the spirits of nitre, frequently taken by teaspoonfuls in mint tea or the patient's common drink, will answer every purpose, and may alone be depended upon," p. 43. " At the

* For an Account of it, see Vol. X. page 78.

the height of this fever then, larger doses of bark in substance, with elixir of vitriol and red wine, were highly useful and necessary," p. 45. Towards the decline, when there were white specks or sloughs about the glands or palate, resembling those of ulcerated sore throats, "these were treated with antiseptic detergent gargles of tinct. myrrh. spir. of vitriol, oxymel simp. and the bark in substance was given internally, with vitriol, in larger or more frequent doses than in any other condition of this disease." p. 48, 49. In the next page comes the consideration of the air, in which is included the first notice of the use of the mineral acids in a state of gas to correct contagion. "The temperature of the air in which the patient breathes must be rendered as favourable to his recovery as possible, throughout the whole course of the disease; for this reason it must be continually renewed, especially when the patient or patients are crowded in small confined places, by opening the doors and windows of the apartments, so as to admit the free passage of the air. This practice, so necessary in the small-pox, is no less so in every species of putrid fever; but it must be done so as not to repress perspiration. The necessity of changing the air in a sick room, by successsive ventilation, arises from the constant destruction of a certain property in that fluid by breathing, which renders it afterwards useless; likewise, from the atmosphere being filled with the excrementitious steams which fly off from the patient's body continually, and which putrify in a stagnant unrenewed air, and render it truly poisonous, a pabulum morbi, rather than of life. The physician will order the room to be kept sweet and clean, and stools and every thing offensive to be removed as soon as possible. If the external air is immoderately cold and wet, the room must be kept warm and dry, and the fumes of amber, myrrh, benzoin and camphire, may be diffused in the room, if sprinkled upon hot-iron. Vinegar may be sprinkled about cold, if the weather is warm; and boiled with myrrh or camphire, an antiseptic steam will arise into the air, which the patient breathes greatly to his advantage. These steams will preserve the air free from putrefaction, and will insinuate themselves by the absorbent vessels of the lungs into the blood-vessels, and will greatly assist in impeding the progress of putrefaction in the fluids. These are the most commodious, if not the most useful, methods of medicating the air the patient breathes; however, those who prefer the mineral acids may order brimstone to be burnt, or may raise the marine acid very easily, by putting a certain quantity of common salt into a vessel kept heated, upon a chafing-dish of coals; if to this a small quantity of oil of vitriol is, from time to time, added, the air will be filled with a thick white acid steam; but both the marine and sulphureous acids must be disengaged at a considerable distance from the patient, otherwise their extreme pungency will be offensive to the lungs." p. 50, 51.

Such

Such is the abstract of the evidence given by our author, on which his father's claim to priority of discovery is rested.

Dr. Smyth, in his Letter to Mr. Wilberforce, gives the following answer to this evidence.

"The preceding passage requires little comment, a fair and candid exposition of the text being all that is necessary to do away the pretended claim. Dr. Johnstone recommends the fumes of amber, benzoin, &c. with the fumes of vinegar, as the effectual means of resisting putrefaction or destroying contagion; and only mentions the mineral acids of sulphur and sea salt, upon a supposition that some persons might choose to employ them. For such persons he gives the common directions how they are to be procured, and a caution respecting the inconvenience or danger that may follow from their use; although his directions on this subject are too general and inaccurate to be of much service."

To this our author replies very fully, and, in our opinion, very satisfactorily evinces his father's claim to the discovery of the power of mineral acids in a state of gas to destroy contagion; but for the arguments and proofs, we must refer our readers to the work itself.

Dr. S. compares other parts of the historical Dissertation with the passage above quoted, and infers, that Dr. J. could not consider the mineral acids as very important, or he would not have dismissed them with so slight and solitary a notice.

This is his first negative argument, and it receives, as it requires, but a short answer from Dr. J.

The second is drawn from the Thesis on the malignant sore throat, published by Dr. James Johnstone, the eldest brother of our author, in 1773, which Dr. S. assumes to have been written by the advice and assistance of his father. In this inaugural Essay, no mention is made of the acid fumigation, and, therefore, Dr. S. concludes, that both father and son, did not think it worthy of public notice. In answering this argument, our author proves Dr. Smyth's assumption to have no foundation in truth; and that the introduction of the subject in an inaugural Thesis, would have been contrary to general usage; but that his brother afterwards, when he re-published his Essay, in English, makes particular mention of this gas as a destroyer of contagion.

It appears also, from a correspondence between Dr. J. and Dr. Priestley, in the year 1775, that both father and son were endeavouring to attract the public attention to this subject in various ways. The testimony of Mr. Crane, a respectable surgeon of Kidderminster is also adduced, in conjunction with many other Extracts of Letters and Details, to shew that Dr. J. senior, and his sons, never lost sight of the importance of acid fumigation in arresting the progress of contagion, from the first discovery in 1756, to the time of Dr. Smyth's publication in 1795. This appears, particularly, by the employment of the fumigation in Dr. Johnstone's own family whenever a contagious fever occurred in it; by by his son Edward's recommending a trial of it to Professor Hope of Edinburgh; by his writing an exercise for his degree, in which, he mantains its efficacy and importance; and by a paper which he wrote for the Medical Society of Edinburgh on the same subject; in all which he mentions it as a discovery of his father, and dwells on the experience both of his father and brother in support of its corrective powers.

When our author has finished his reply to Dr. S. on the subject of the priority of his father's discovery, he enters into an examination of Dr. Smyth's claim, and particularly of the period, when Dr. S. had actually made up his mind respecting the superiority of the nitrous over the muriatic fumigation in a state of gas. This, as far as can be collected from circumstances, does not appear to have been earlier than 1794 or 1795. Upon the whole, we think that Dr. J. has vindicated his father's claim to priority of discovery, and his own character for accuracy and candour, against Dr. Smyth's harsh attack, with a firmness and moderation no less creditable to himself, than honourable to the cause he supports.

---

MEMOIRS *of the* MEDICAL SOCIETY *of* LONDON, *instituted in the Year* 1773. Vol. VI. 8vo. London.

"Article I.—*Sketch of the similarity of ancient and modern Opinions, respecting the Morbus Cardiacus*; by WILLIAM FALCONER, M. D. F. R. S."

That a difeafe in the times of the ancient writers, fhould exhibit fymptoms fimilar to thofe obferved in the prefent days, is not very furprifing: but we really felt fomewhat amazed, when we found a complaint, hitherto confounded with fyncope, was the identical difeafe defcribed by Huxham, under the name of *febris cuta nervofa*; by Savage, as *Typhus nervofa*; by Cullen, as *Typhus mitior*; and by Wall as low fever. This, Dr. Falconer conceives to be the difeafe called by Aretæus and Galen Νοσος καρδιακος, and by Celfus, Pliny, and Cælius Arelianus, Morbus Cardiacus; and remarks, that the mode of treatment has been varied by the ancients and the moderns in a fimilar manner. Of the mode of treatment by the mathodic fect, Cælius Arelianus contains moft that we now know. It is eafy to learn from him, that their remedies being few, the fame were neceffarily applied to a variety of complaints, and this more particularly, as they undertook to reduce difeafes to certain claffes, which, of courfe, induced them to generalise the method of treatment. It is therefore no wonder, if among thofe he propofes, Dr. Falconer fhould find many which are ufed by modern practitioners for typhus. Yet many, even of the remedies propofed by Aretæus and Aurelian, we apprehend, would be of little confequence in typhus, though we fhall prefently fhow that they are very ufeful in

 the the difeafe for which thofe writers propofed them. To what purpofe fhould we expofe to the view of a patient, under typhus, flowery lawns, meadows, and foftly flowing ftreams? why attempt to refrefh him with oderiferous gales? When the fenfes are fo hebetated that he becomes indifferent to the moft interefting occurrences in his own concerns, what relief is he to feel from fcenes which are only grateful in proportion as the mind is in a condition to enjoy them?

Whoever ftudies attentively the writings of the ancients, will perceive that morbus cardiacus is in all, more or lefs, connected with fyncope, as we now ufe the latter term Aretæus evidently diftinguifhes two fpecies, the moft formidable of which, is that which is the termination of καυσος. This latter difeafe appears to have been the ardent fever of warm climates, which at certain feafons, runs through its periods with fuch celerity as to finifh, after a few exacebartions, and even after a fingle one in a fyncope, from which the patient never recovers. On this account, that cautious and elegant writer very properly teaches us, that our bufinefs is, in thefe cafes, to prevent fyncope, or morbus cardiacus, rather than to cure it. When we apprehend it therefore from the prefence of καυσος, we fhould bleed in the arm, unlefs fome very ftrong objection occurs. We may likewife draw blood by fcarification and cupping, left we fhould fink our patient too faft, always recollecting the weaknefs which is to follow the violent action from *caufus*. When the difeafe (*morbus cardiacus*) is prefent, all thofe means are to be ufed which may reftore the ftrength, and if poffible fupport life. If afterwards that languor remains, which is the frequent confequence of ardent fever; or, if from any other caufe, the patient fhould be fubject to fainting fits, attended with a low ftate of health and dejection of fpirits, all the means are to be ufed which are likely to reftore his ftrength, recruit his fpirits, and banifh from his mind every gloomy reflexion or unpleafant idea.

Tho' Aurelian in this, and every other part of his writings, is more confufed than Aretæus; yet it is eafy to perceive in both a diftinction between the treatment during the ftate of fyncope and thofe intervals, in which attention was to be paid to prevent the return of the fit. But we are exceedingly at a lofs to know why the learned author of the paper, whilft he is defcribing the difeafe from the ancient writers, fhould prefer that part of Aretæus which relates to the treatment, and entirely pafs over the caufes and figns. This is the more remarkable, as one divifion of the paper is on the caufes of the difeafe affigned by the ancients. This omiffion is fo unaccountable, that to a lefs learned writer, we fhould be difpofed to make feveral remarks, which we need only hint to Dr. Falconer. In the αιτιαι και σημεια that gentleman will at once recollect, that the fame difeafe is called fyncope, and defcribed as the moft ferious confequence of the ardent fever. In addition to the fymptoms he has enumerated in the paper, he will find feveral others; particularly, that the patient, during the *caufus*, breathed fire, but that in the fyncope ftage his breathing is cold; when arrived rived at this ſtate, it is unneceſſary to add, that the caſe muſt prove fatal. It is therefore no wonder if Aretæus conſiders bleeding as fatal in ſyncope, though he adviſes it warmly in the early ſtage of *cauſus*, in order to prevent the future dangerous termination. He marks the progreſs of the two ſtages with his uſual warmth of expreſſion, obſerving, that the heat is converted into cold, and dryneſs into a ſhower [of perſpiration]. The mind too, he obſerves, not only recovers itſelf from the delirium, which is a ſymptom of *cauſus*, but is in the syncope more acute than in health. This refers to that compoſure which all the phyſicians of the ſouth deſcribe, as ſo remarkable in the laſt ſtage of ardent fever, and which anciently gave riſe to the ſuperſtitious notion, that dying men could prophecy. It may even be ſuppoſed that the word ſyncope was either the cauſe or the conſequence of this error, *συνκοπη παρα το συνκοπτειν a concidendo utpote quæ nexus animæ et corporis ſubito, abſcindat*, as the learned Gregorius Magnus, and after him Petit remarks. By the ſudden failure of all the corporeal powers, whilſt the mind retained its energy, it was conceived that the ſeparation between ſoul and body had already taken place, which enabled the patient to converſe with the dead, and to learn many particulars, which he ſtill retained the power of communicating in the ordinary way.

Such is Aretæus's account of ſyncope, or *morbus cardiacus*. Aurelian is more confuſed, but in every part of his deſcription refers to fainting fits. Even the quotations produced by the learned author of the paper, concerning the cauſes to which Aurelian and Galen imputed the diſeaſe, are all ſuch as we ſhould expect to produce ſyncope.

Of Pliny's account of this or any other diſeaſe we know but little. He was ignorant of what medical knowledge was current, even in his own days, and only picked up remedies and receipts wherever he could procure them. However, by his propoſing the ſame remedy for lethargy and morbus cardiacus, there is reaſon to believe he had ſome view to fainting, or a ſudden incapacity of muſcular exertion. Galen's account does not materially differ.

It is evident that Celſus knew but little of the *καυσος* of the Greek Aſiatics. However, his arrangement places morbus cardiacus among thoſe diſeaſes, qui huic (febrium curationi) ſuperveniunt. His whole deſcription leads to that kind of fainting, in which the ſenſes are not alienated; for whenever a perſon who faints, is in a condition to expreſs his feelings, he never ſhows any abſence of mind. The remedies propoſed by this accurate writer are, firſt to relieve the immediate ſymptoms, and as much as poſſible to repreſs the ſweating, which were conceived to add much to the general weakneſs. For the firſt purpoſe, the ſame remedies were uſed as obtain to the preſent day, and muſt for ever obtain. The patient, whilſt he fainted, was to have freſh air. He was alſo to be rubbed; a remedy highly important, though at preſent too often neglected. In order to reſtore the ſtrength, and prevent a return of ſuch faintings, the patient is directed to be well and carefully fed; and every other means were to be ufed, which might reftore his ftrength.

We have dwelt longer on this article than its importance entitles it to, becaufe we conceive it particularly the bufinefs of a Reviewer to examine carefully the authorities which are quoted; and in doing this, he can fcarcely fail to impart fome of his enquiries to his readers. Befides which, we conceive it a juftice we owe Dr. Falconer, thus to explain our reafons for differing from him, as from the nature of our work, he will have an opportunity of explaining himfelf further. In faying this, we are not courting a controverfy, which muft prove unprofitable to our readers, and irkfome to ourfelves; but only apologifing for the length of our article.

"Article 2.—*A Cafe of Angina Pectoris, with a Diffection.* By Samuel Black, M. D. of Newry, Ireland."

Though this difeafe is by no means uncommon, the above cafe is extremely well worth recording, on account of the great relief the patient found from iffues, and particularly from his lofing all thofe advantages, by drying up his iffues, which, though they were afterwards opened, did not produce the fame good effects. The fufpending a difeafed action, by promoting a new and diftant one, is always uncertain. But if we have the good fortune to fucceed, it is ftill more doubtful, whether our new action will become habitually fufficient to prevent the return of the old. If ever we find fuch a confequence, we fhould not fail to avail ourfelves of it. We ought in juftice to add, that the *patient only* appears to have been anfwerable for the confequence of drying up the iffues.

"Article 3.—*Cafe of Hydrocephalus Internus, terminating fuccefsfully.* By Edmond Pitts Gapper, Surgeon at Ewell, Surry."

This is a very valuable cafe, but is like too many others, not related with fufficient minutenefs. By the general hiftory we fhould fufpect that the difeafe originated in *coup de foleil*, which at firft produced extravafation of lymph from increafed action, and that this increafed action afterwards became habitual, returning at uncertain and fhortened intervals. The cafe, however, being as we before remarked, related in too few words, we fhall fubmit them to the reader, leaving him to form his own judgement.

"The fubject of this cafe, is a girl of twelve years of age, whom I was defired to vifit, fome time in January laft. She was feized in the fields, while employed in picking ftones, with univerfal pain, heavinefs in her head, and dimnefs of fight. This laft fymptom I was not informed of at the time, and could get no other account from her, than that "fhe was ill all over." At this time there was not the leaft heat on her fkin, or any fymptom of fever; the pulfe was fcarce perceptible, and fhe appeared ftupid and infenfible; fome gently opening medicines were ordered, and I faw her again in a few days, my affiftant having vifited her in the interval. I then found her complaining of ficknefs, and pain in her head, though not in a fufficient degree to excite in me an idea of there being any thing thing particularly extraordinary in the caſe. An emetic was adminiſtered, a bliſter applied to the nape of the neck, and the doſes of opening medicines increaſed, as coſtiveneſs was much complained of. She went on in this manner near a week, when all the ſymptoms began to increaſe; the pain in her head became ſo acute, as to produce at times, actions of violence, which rendered confinement neceſſary, and the intervals were marked by ſtupidity; ſhe was totally unable to bear an erect poſture, and her diſtreſs was very great and affecting. Theſe circumſtances led to a more particular examination. I found the pupil of the eye dilated to its utmoſt extent, and abſolutely immoveable, the pulſe ſmall and extremely quick, with a dry hot ſkin, a conſiderable degree of thirſt, though the tongue was but ſlightly furred. A ſtupor, conſtant watchfulneſs, a ſenſe of great weight in the head, ſickneſs, obſtinate coſtiveneſs, and ſmall evacuations of urine, were prominent ſymptoms, and which led me to infer, that the diſeaſe in queſtion could be no other than Hydrocephalus Internus."

The author's treatment was very judicious, as the event proved. Mercurial frictions produced ſalivations, and a diſcharge from the noſe. By this courſe the returns of inflammation ſeem to have been prevented, but delirium ſtill remained, probably, becauſe all the extravaſated fluid was not abſorbed. Nothing could be better calculated to complete the cure, than Mr. Gapper's plan of bliſters to the head, and occaſional vomits: with theſe he ſucceeded.

"Article 4. — *Caſe of a Boy who became of a blue Colour ſome Months after Birth.* By Edward Thomas, M. D. &c."

This is an intereſting caſe, leading indeed to no practical inferences, but containing a very good caution againſt haſty prognoſis, and particularly againſt undervaluing the accounts of extra-profeſſional relators.

"Article 5. — *A Caſe of obſtinate Hepatic Diſeaſe.* By J. C. Lettsom, M. D. &c."

This caſe is curious in many reſpects, but the *denouement*, to ſay the leaſt of it, is not miraculous. The patient, being a medical man, had of courſe, his choice of phyſicians without expence, and could judge of the effects their remedies might produce. After uſing them all without benefit, he was attacked with acute ſymptoms, threatening fatal conſequences, but ending in a complete and apparently permanent cure of his chronic complaints.

"Article 6. — *Caſe of a remarkable and ſucceſsful Termination of Scrotal Hernia.* By James Lee, Surgeon, Spaniſh Town, Jamaica."

This is another caſe relieved by the ſame phyſician as the laſt. The hiſtory, however, is not leſs inſtructing, and ſuch hiſtories do the relators particular credit, not only as they derive no very ſplendid reputation from them, but becauſe they evince gratitude to a benefactor, to whom the patient and phyſician are often ſo much indebted. During the operation the parts were found gangrenous, ſo that an artificial anus became neceſſary. About a year afterwards afterwards the patient was feized with acute pain, attended with a diminution of the orifice from adhefion, ending in expulfion of the fæces per anum, and a complete cicatrization of the wound.

"Article 7. — *A Cafe of Croup fuccefsfully treated by Emetics.* By JOHN SMITH, Surgeon, C. M. S. and Member of the Medical Society of Philadelphia."

"Article 8.—*Cafe of Opifthotonos fuccefsfully treated.* By the fame."

The author in this cafe applied a cauftic to a sore on the leg, which had scabbed, and from which he conceived the difeafe arofe. To this, with the free ufe of fal. chalybis and flor. zinci, he imputes the cure.

"Article 9. — *On the Origin of the Cow-Pox.* By JOSEPH HEAD MARSHALL, M. D."

We fhall tranfcribe the whole of this paper, becaufe we conceive the fact recorded in it cannot receive too great publicity.

"The very extraordinary affertion brought forward by Dr. Jenner, in his firft publication on the cow-pox, namely, that that difeafe originates in the horfe, and not in the cow, has occafioned much controverfy. The following fimple detail of a fact, that very lately came under my own obfervation, will, I think, tend greatly to elucidate it.

"Being called to vifit a young woman, who is a dairy maid at a farmer's, I found her in bed, complaining of a pain in her back, laffitude, and thirft. Her face was flufhed, and her tongue foul. Upon requefting her to give me her arm, I difcovered upon the hand four or five large puftules, which, from my knowledge of the difeafe, I immediately afcertained to be cow-pox. On the back of the hand there had evidently been a long fcratch, on a part of which appeared the primary puftule; the others were very near it.

"Upon making a ftrict enquiry, I found one of the cows had this difeafe, and that in feveral of the others, it was alfo advancing. On farther enquiry, I alfo found, that the farmer had a horfe with fore heels in the ftable, which his fon always attended, who did not ufually milk the cows; but that one morning, this cow being troublefome and reftive, he had, to relieve the dairy-maid, milked her himfelf.

"From this plain and fimple ftate of facts, the origin of the difeafe became, in this inftance, very apparent, as it could be clearly traced from the horfe to that individul cow which the young man milked, and through this medium to the dairy-maid, and thence to the reft of the herd."

"Article 10. — *Cafe of Framboesia Guineaenfis, or Yaws.* By JOSEPH ADAMS, M. D. &c. of the Island of Madeira."

It is some satisfaction to observe a coincidence in the accounts of medical men, where we cannot suspect any communication between them. This paper, if it was not read before the Society, muft must have been written prior to the publication of Dr. Winterbottom's Account of African Diseafes; yet there is no probability that the Doctor could have seen it. However, in the most important part of this disease, as to its symptoms, the two accounts agree. Dr. Adams's, it will be supposed, is the most minute; as his attention to all the discriminating symptoms of morbid poifons is well known. The cafe being long, and not admitting any curtailment, we shall only transcribe some of the author's remarks, by which it will appear that Frambœsia, though placed by Cullen under the order *impetigines*, fhould, according to his fyftem, have been arranged with the exanthemata. This is no imputation against that celebrated nofologift, becaufe he himfelf confeffes *de elephantiafi, lepra, frambœfia, et trychomate utpote a me nunquam vifis amplius ftatuere non aufus fum.* It further appears by the account before us, that tho' frambœfia, by paffing through its ftages and arriving at a natural cure, refembles variola and the other exanthemata, yet in its flow progrefs it muft often affume the form of impetigines.

"Having thus defcribed the character of the difeafe, I fhall offer a hiftory of it, founded on obfervation, and leading to practice.

"1ft. The violence of the difeafe must be in proportion to the fufceptibility of the conftitution for it.

"2d. When the fufceptibility is great, it is likely to be proportionably permanent; and as long as it continues, the matter of each puftule will infect thofe parts it comes in contact with. Hence the fpreading of the fame individual puftule or fcab.

"3d That nothing will deftroy the fufceptibility of a part, or the whole conftitution, but its full action. Hence,

"4thly. Though the action may be fufpended for a time, by exciting a different action, yet, the fufceptibility ftill remaining, the action will return as foon as that which fuperfeded it ceafes.

"To illuftrate this theory the better, I fhall contraft yaws with the only two morbid poifons to which it bears any analogy, without exactly refembling either.

"The venereal is a poifon of which the conftitution is for ever fufceptible, and which it has no power of curing in itfelf: confequently, the matter from every ulcer affects the contiguous parts, and the difeafe is kept up for ever, or till a more powerful ftimulus is applied; after which, on a frefh application of the infection, the conftitution is found as fufceptible as before.

"The fmall-pox is a poifon of which the conftitution is no longer fufceptible, after having gone through a certain fever and eruption, occafioned by the application of its poifon. Confequently, from this time, the contiguous parts being infenfible to the variolous irritation, all the puftules heal without fpreading, and the conftitution is found to have loft its fufceptibility on a frefh expofure to the infection.

"Of yaws, on the contrary, the conftitution remains fufceptible after the eruption and fever (if any happen to attend it) are completed.

pleted. Hence, as in the venereal, the pus affects the contiguous parts. But this fusceptibility only continues for a time, uncertain according to the difference of conftitution, or ftate of it at the time. When the fusceptibility ceafes, the parts heal as in the fmall-pox, though more flowly, from the flow progrefs of all the other actions. When healed, the conftitution has for ever loft its fusceptibility for the difeafe.

" In one point they all agree, namely, that they may be fufpended for a time by another more powerful ftimulus, but will fhow themfelves as foon as the effect of that ftimulus ceafes. When it happens that the conftitution is infected by abforption from a local venereal ulcer, the confequent difeafe will never fhow itfelf while the mercurial irritation which cured the chancre continues; but when that irritation ceafes, the difeafe will appear in the fkin, fauces, or bones.

" In the fmall-pox, inoculation has taught us, that, after the infection is received by a conftitution fufceptible of its impreffion, the difeafe may be fuperfeded by fome other irritation, moft commonly an eryfipelatous fever, or the meafles. As foon, however, as thefe ceafe, the fmall-pox refumes its action, and continues its courfe.

" In yaws, the progrefs of which is particularly flow, it appears that, even after the difeafe has proceeded to fuppuration, it may be fuperfeded for a time by mercury. But if that remedy has been applied before the full action of the difeafe, namely, fcabbing, has taken place, whenever the mercurial irritation ceafes, the yaws refume their action, which continues as long as the conftitutional fufceptibility remains.

" To conclude, the conftitution is always fufceptible of the venereal poifon; fo that the difeafe will fpread till fuperfeded by a more violent irritation; and return on a frefh application of the poifon. The fmall-pox will cure itfelf as foon as the fuppurative fever is over; and with the difeafe, the fufceptibility for it is extinguifhed for ever.

" In yaws the fuppuration, whether attended with fever or not, does not immediately relieve the conftitution from its fufceptibility to the difeafe: nor is there any remedy yet known that will cure it. But this fufceptibility ceafes by degrees, after which the parts heal, and the fufceptibility never returns.

" Though only the venereal is abfolutely incurable, excepting by a remedy, yet all three may be arrefted, at certain ftages, without being cured.

" That fuch is the nature of yaws, appears from every authority I have been able to find, and from my own obfervation. The anonymous and modeft author of a paper in the Edinburgh Medical Effays, Dr. Hillary, and Sauvage, all agree, that if mercury is given before all the yaws are fcabbed over, the beft that can happen is a return of the difeafe when the mercurial irritation ceafes; but all of them, as well as Mr. Hunter, mention very calamitous events that have fometimes followed the early exhibition of of mercury. The two first mentioned writers (the only two practical ones) never feem to have left the difeafe to its natural cure, and differ much in their opinion of fuch a practice. Hillary, with his ufual haftinefs, conceives it would always kill the patient; but the other has the modesty to believe it would probably get well of itfelf, though he never had the courage to try. Both agree that, after falivation, fome of the yaws will obftinately refift, and that it will be neceffary to rub them with cauftic to the bottom."

The article concludes with an enquiry, whether this difeafe is the fame as the leprofy of the Jews? It appears, by a variety of arguments, that no other difeafe has ever been defcribed, or is now known, which refembles, in all its forms, the leprosy defcribed in Leviticus. Dr. A. conceives the doubts on the fubject have arifen from confounding this leprofy with another which afterwards occurs in holy writ, and which being evidently incurable either by the common powers of the conftitution, or any known remedy, became a proper object of the difplay of the miraculous power.—Such was the leprofy of Naaman the Affyrian, of king Uzziah, and feveral others. Had this leprofy been the fame for which a temporary retirement was required, after which the fubject re-admitted into the congregation, it would not have been worthy of a miracle either in its cure or infliction.

Article 11.—*Cafe of Extra Uterine Fœtus*; by A. FOTHERGILL, M. D. F. R. S. &c.

This cafe terminated as unhappily as many of the fame kind. The hiftory is worth recording, but can afford no new practical hints under fimilar events. The author has, however, added fome remarks, which may be perufed with advantage on the propriety of extracting fuch fœtufes by an incifion through the parietes of the abdomen. If we can depend on all the fuccefsful hiftories related by the French furgeons, we can only wifh that our own had more courage and equal fuccefs: for if thefe incifions into the abdomen can be made with fafety, it would be very defireable to attempt them in cafes of cyftic dropfies, whether from the ovaria or hydatids.

Article 12.—*Cafe of Inverted Uterus after Parturition.* By THEOPHILUS DYSON, Surgeon, F. M. S.

In this cafe Mr. Dyfon was fuccefsful in replacing the uterus in its natural form, after which no further ill confequences occurred. We are informed his patient has fince been delivered of another child, the uterus retaining its natural fituation.

Article 13.—*An Account of an extraordinary Mafs of Difeafe formed in the left Cavity of the Thorax.* By J. CARDEN, Surgeon to the Worcefter Infirmary.

This is a very interefting cafe, well related, and followed by very judicious remarks.

Article 14.—*Hiſtory and Diſſection of a Caſe of Inteſtinal Ulceration, with Remarks.* By Henry Field, Sec. M. S.

This caſe ſhews, like many others, the imperfect knowledge we can form of inteſtinal diſeaſes, by the ſymptoms and deſcription of the patient. The hiſtory and diſſection are well related, and the remarks by the author judicious.

Article 15.—*Hiſtory of a peculiar morbid Appearance in the Heart.* By James Hume Spry, Member of the Royal College of Surgeons, &c. F. M. S.

This hiſtory is certainly curious, and well worth recording; we are only ſurprized that the committee of papers ſhould not have pruned off ſome of the ſentences. In this, our profeſſional readers will agree with us, unleſs they have ſo far forgotten their early education as to be ignorant of the fœtal circulation, and of the properties acquired by the blood during its circulation through the lungs. We admit it is well worth recording, that the foramen ovale and canalis arterioſus, not only remained pervious, but ſeemed to increaſe like the other parts of the body as long as the ſubject lived, which was to the age of ſeventeen. The ſymptoms were ſuch as might be expected, laborious reſpiration, incapacity of exertion, and a blue colour occaſionally, amounting in the extremities almoſt to black.

The other papers of this volume muſt be deferred to a future Number.

